# A re-irradiation dose of 55–60 Gy improves the survival rate of patients with local recurrent esophageal squamous cell carcinoma after radiotherapy

**DOI:** 10.1186/s13014-021-01828-z

**Published:** 2021-06-08

**Authors:** Xun Wu, Xingsheng Hu, Junru Chen, Lang He

**Affiliations:** 1 The First School of Clinical Medicine, Lanzhou University, Lanzhou, 730000 Gansu People’s Republic of China; 2 Department of Oncology, Chengdu Fifth People’s Hospital, No 33, Mashi Street, Wenjiang District, Chengdu, 611130 Sichuan People’s Republic of China; 3 Department of Oncology, The Second Xiangya Hospital of Central South University, Changsha, 410000 Hunan People’s Republic of China

**Keywords:** Esophageal squamous cell carcinoma, Recurrence, Salvage chemoradiation therapy, Salvage radiation therapy

## Abstract

**Introduction:**

Local recurrence (LR) is clinical challenge in the treatment of esophageal squamous cell carcinoma (ESCC). The current study aimed to determine the optimal re-irradiation dose for local recurrent esophageal squamous cell carcinoma (LRESCC) following radical (chemo) radiotherapy.

**Methods:**

We retrospectively analyzed 125 patients with LRESCC after receiving initial radiotherapy. For radiotherapy treatment, 58 patients were assigned to low-dose (LD) group (50–54 Gy) and 67 were assigned to the high-dose (HD) group (55–60 Gy). The response rate (complete + partial response), 1-, 2- and 3-year survival rate, and toxicity were recorded. We then analyzed the impact of different radiotherapy doses and combination chemotherapy on the survival of patients with LRESCC.

**Results:**

After re-irradiation, the 1-, 2- and 3-year survival rates in the LD and HD groups were 48.3%, 24.1% and 10.3% and 61.2%, 34.3% and 19.4% in the HD group, respectively, and the difference in overall survival rate between the two groups were significant (*P* < 0.05). The median survival time of patients receiving radiotherapy alone was 9 months in the LD group and 15 months in the HD group (*P* < 0.05). The survival rate of patients treated with chemoradiotherapy was higher than that of patients treated with radiotherapy alone in the LD group. However, chemoradiotherapy showed no advantage over radiotherapy alone in the HD group. In addition, the incidence of radiation esophagitis, the most common toxicity, was higher in the HD group compared to the LD group (68.7% vs 58.6%). Multivariate analysis demonstrated that re-irradiation dose was an independent favorable prognostic factor in patients with LRESCC.

**Conclusion:**

Higher re-irradiation dose (55–60 Gy) can improve the long-term survival of patients with LRESCC after radiotherapy, with tolerable toxicity.

## Introduction

Esophageal squamous cell carcinoma (ESCC) is the most common esophageal malignancy reported in China. Mostly radiotherapy and surgery are considered as the mainstay treatments for ESCC. Unfortunately, the patients with ESCC undergoing radiotherapy the local recurrence rate is found to be 40–60% [1]. Moreover, without active intervention, most patients experience disease progression and die within 1 year [2, 3]. To date, there is no consensus on the optimal treatment of local recurrency of ESCC (LRESCC) following (chemo) radiotherapy. Previous studies have shown that salvage surgery can effectively treat LRESCC patients after radiation therapy [4–8]. However, fibrosis of tissues surrounding the tumor bed after radiotherapy complicates the surgical intervention and increases preoperative risk, even in carefully selected patients [9, 10]. Recently Chen et al*.* has reported that re-irradiation therapy can achieve similar survival outcomes as that of salvage surgery [2]. Hence, re-radiotherapy may be an important salvage treatment for patients with LRESCC [2, 11]. It is also noted that the dose of radiotherapy can influence the survival outcomes [12]. For a better survival the salvage radiation dose should be between > 50 Gy and < 60 Gy for LRESCC patients [13, 14]. However, whether higher doses of re-irradiation, between 50 and 60 Gy, would be more beneficial to patients with LRESCC after initial radiation remain to be resolved. This study we investigated the survival rate and toxicity of different re-irradiation doses combined with or without chemotherapy in the treatment of LRESCC.

## Patients and methods

### Patients and clinical features

We retrospectively selected 207 patients with LRESCC who underwent re-irradiation therapy in the Chengdu Fifth People’s Hospital between January 2012 and December 2016. Patients with a non-recurrence with interval of > 6 months after initial radiotherapy, confirmed (pathological examination, imaging or gastroscopy) recurrence of primary esophageal cancer with or without local lymph node metastasis, or only local lymph node metastasis (supraclavicular fossa, mediastinal, esophageal, or para-aortic lymph nodes). The re-irradiation therapy dose was 50–60 Gy, as well as those with the Eastern Cooperative Oncology Group (ECOG) score 0–2, with functional heart, liver, kidneys, lungs, and bone marrow hematopoiesis were enrolled in the study. We excluded patients with distant organ metastasis, tumor in other organs, or those with incomplete information. Patients who received hyper-fractionated radiotherapy and surgical resection for esophageal cancer were also excluded. Finally, 125 patients were included, 84 men and 41 women with a median age of 68 years (range 50–89 years). All the patients had either refused surgical intervention or were unable to undergo salvage surgery. The selected patients showed locally recurring tumors were located in the previously irradiated area. The study was approved by the Ethics Committee of Chengdu Fifth People’s Hospital.

### Treatment and follow-up

A total of 125 patients were initially treated with two-dimensional radiotherapy, three-dimensional conformal radiotherapy (3D-CRT) or intensity modulated radiotherapy (IMRT), with a median dose of 60 Gy (50–66 Gy). Most of the patients received chemotherapy regimens containing cisplatin, paclitaxel or fluorouracil. After the initial treatment, gastroscopy, esophageal barium meal radiography or enhanced computed tomography (CT) was performed to evaluate the efficacy of the treatments. All patients achieved either complete or partial response.

The patients were subjected to re-irradiation with 3D-CRT or IMRT at a dose of 1.8–2.0 Gy per fraction, 5 days/week. Gross tumor volume (GTV) was assessed using esophageal barium meal examination and CT images. GTV for metastatic lymph nodes (GTVnd) revealed enlarged lymph nodes with short diameter > 10 mm. The examination using positron emission tomography–CT indicated the presence of metastatic lymph nodes. Planning target volume (PTV) was performed by extending 0.5–1.0 cm radially from the GTV, and 1.0–2.0 cm above and below the GTV, or 0.5 cm outside the GTVnd in all directions. The lymphatic drainage area was not prophylactically irradiated. Dose limitation for normal tissues or organs was: bilateral lungs V20 < 25%, spinal cord Dmax < 20 Gy, and mean radiation dose to the heart < 30 Gy. As a variety of dose fractionation schedules were employed, the biological effectiveness of radiation schedule was calculated by the biologically effective dose (BED) formula: BED = n × d (1 + d/(α/β)), where n is the total number of fractions, d is the dose per fraction and α/β is the alpha/beta ratio of the organs at risk (α/β = 3 Gy). In the middle of the re-radiation, all patients received a chest CT scan to assess the condition of the lungs.

The overall survival (OS) was defined as time from the beginning of retreatment until last follow-up or patient death. Efficacy was evaluated at the end of retreatment and 1 month after the end of retreatment. Follow-up evaluations were conducted every 2–3 months for the first year, 3–4 months the second year, and 6 months thereafter. Evaluation methods included clinical examination, endoscopy, tumor markers, esophageal barium meal, or enhanced CT. Toxicities that occurred within three months after the initiation of re-irradiation were defined as acute toxicities, whereas late toxicities were defined as those first observed three months after or those lasting for > 3 months after the initiation of re-irradiation. The response rate (RR) was defined as the percentage of patients with complete response and partial response in the total number of cases. Therapeutic responses were evaluated using response evaluation criteria in solid tumors (RECIST 1.1), acute and late toxicity was graded in reference to the Common Terminology Criteria for Adverse Events (CTCAE v4.0). According to the re-irradiation dose, 58 patients were assigned to the low-dose (LD) group (50–54 Gy, median dose 50 Gy) and 67 patients were assigned to the high-dose (HD) group (55–60 Gy, median dose 60 Gy).

### Statistical analysis

Data analysis was performed using SPSS version 23.0 (IBM Corporation, Armonk, NY, USA). Continuous or categorical variables in the two treatment groups were compared using the Student’s *t*-test or the chi-square test. The survival rates were calculated by Kaplan–Meier method, and the difference in survival curves was compared by log-rank method. Cox’s proportional hazards regression model was used to determine the effect of univariate or multiple factors on the survival rate. Time to recurrence (TR) was defined as the time between the beginning of initial treatment and confirmation of recurrence. Two-sided *P* < 0.05 was considered to be statistically significant.

## Results

The clinical baseline characteristics of patients in the two groups are shown in Table 1. A total of 67 cases of primary recurrence (PR), 33 cases of PR with local lymph node recurrence (PR + LNR) and 25 cases of local lymph node recurrence (LNR) after initial radiotherapy were recorded. Sixty-eight patients which is of 54.4% relapsed within 2 years following initial definitive radiotherapy. The median time to recurrence in all patients was 21 months (range 8–201 months). After re-irradiation, the median follow-up time was 19 months (range 4–65 months). The patients who lived in the period of time were followed up for 40 to 65 months (median, 47 months). At the end of the follow-up, 2 patients survived in the LD group, while 7 survived in the HD group. Total 3 patients were lost to follow-up. A total of 102 patients received 3D-CRT or IMRT during the initial and re-irradiation. Median total radiation dose (BED) of heart, lungs and spinal cord of these 102 patients was 36.5 Gy (range 16.7–98.3 Gy), 95.2 Gy (range 76.1–236.2 Gy) and 65.8 Gy (range 56.6–82.6 Gy). Sixty-one of the 125 patients received 1–4 cycles of concurrent or sequential chemotherapy combined with re-irradiation, which included either single or double-drug regimens containing paclitaxel, platinum or fluorouracil. In the LD group, 8 patients received platinum-containing dual-drug sequential chemotherapy, whereas 17 received single-drug fluorouracil or platinum-containing dual-drug concurrent chemotherapy. In HD group, 14 patients received platinum-containing dual-drug sequential chemotherapy, whereas 22 patients received single-drug fluorouracil or platinum-containing dual-drug concurrent chemotherapy.

**Table 1:** Characteristics of all patients (n = 125)

Characteristics	LD group (%)	HD group (%)	χ^2^/t	*P*
*Gender*				
Male	35 (60.3)	49 (73.1)	2.307	0.129
Female	23 (39.7)	18 (26.9)		
*Age*				
Mean ± SD	69.34 ± 8.13	68.18 ± 7.53	0.832	0.407
*ECOG*				
0–1	48 (82.8)	54 (80.6)	0.097	0.756
2	10 (17.2)	13 (19.4)		
*Recurrence pattern*				
PR	35 (60.3)	32 (47.8)	1.614	0.446
LNR	11 (19.0)	14 (20.9)		
PR + LNR	12 (20.7)	21 (31.3)		
*Initial radiation dose (Gy)*				
Mean dose	58.69 ± 3.10	59.22 ± 2.76	1.01	0.310
*Time to recurrence (months)*				
≤ 24	36 (62.1)	32 (47.8)	2.57	0.109
> 24	22 (37.9)	35 (52.2)		
*Re-radiotherapy technology*				
3D-CRT	25 (43.1)	34 (50.7)	0.73	0.393
IMRT	33 (56.9)	33 (49.3)		
*Chemotherapy*				
Yes	25 (43.1)	36 (53.7)	1.405	0.236
No	33 (56.9)	31 (46.3)		

*SD* standard deviation, *ECOG* Eastern Cooperative Oncology Group Performance Status, *PR* primary recurrence, *LNR* local lymph node recurrence, *3D-CRT* three-dimensional conformal radiotherapy, *IMRT* intensity modulated radiotherapy

### Treatment outcomes

**Fig. 1:**
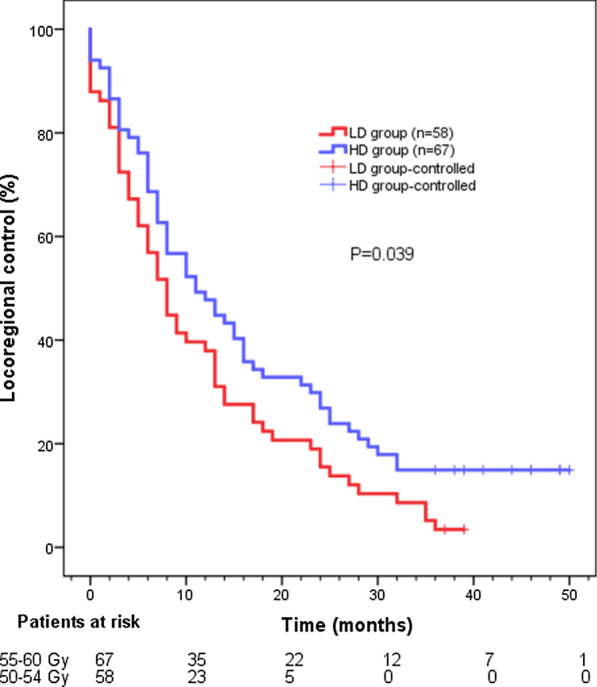
Kaplan–Meier estimates of locoregional control rate for LD group and HD group

**Fig. 2:**
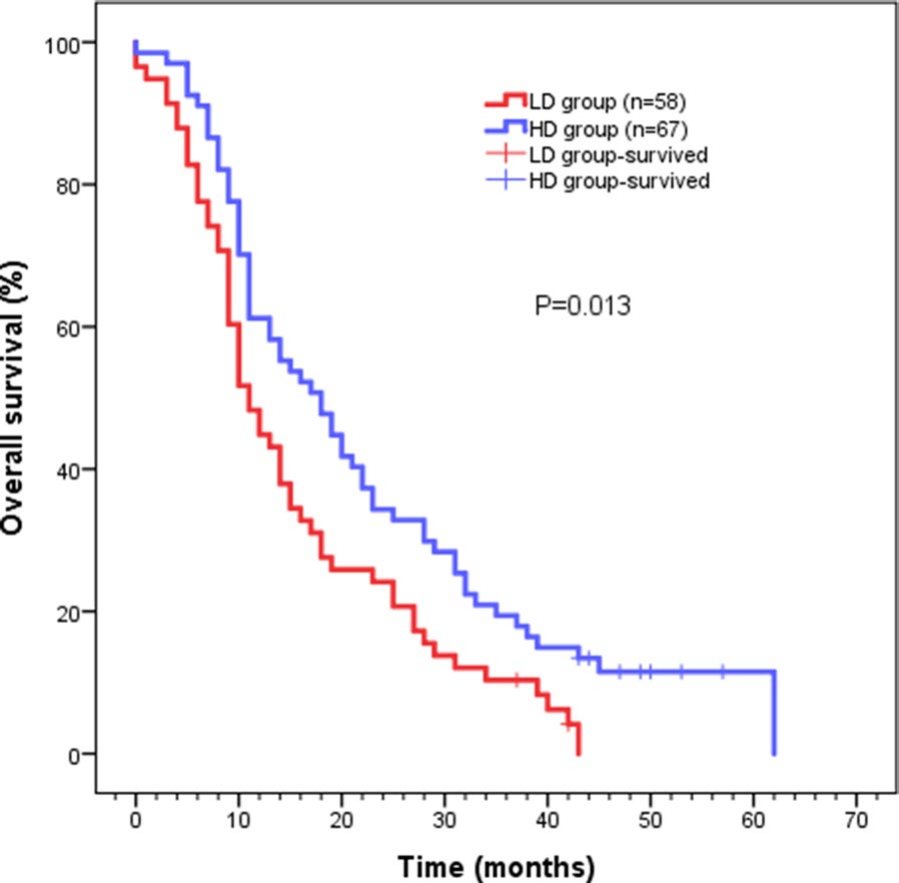
Kaplan–Meier survival analysis for LD group and HD group

After retreatment, the RR was found to be 87.9% (58/66) and 76.8% (43/56) in the HD and LD groups, respectively (*P* = 0.065). The 1-, 2- or 3-year locoregional control rate in the LD and HD groups was 37.9%, 15.5%, 3.4% and 47.8%, 26.9%, 14.9%, respectively. The median time to locoregional recurrence in the LD and HD groups was 8 months and 11 months, respectively (*P* = 0.039) (Fig. 1). The results showed that the 1-, 2- or 3-year survival rate for the whole cohort was 56.8%, 29.6% or 15.2% respectively, with a median survival time of 14 months. The 1-, 2- or 3-year survival rate in the LD group was 48.3%, 24.1% or 10.3%, respectively, with a median survival time of 11 months (95% confidence interval (CI) 8.514–13.486). The 1-, 2- or 3-year survival rate in the HD group was 61.2%, 34.3% or 19.4%, respectively, with the median survival time of 18 months (95% CI 12.276–23.724). Compared to patients in the LD group, those in the HD group had a better higher survival rate (*P* = 0.013) (Fig. 2).

**Fig. 3:**
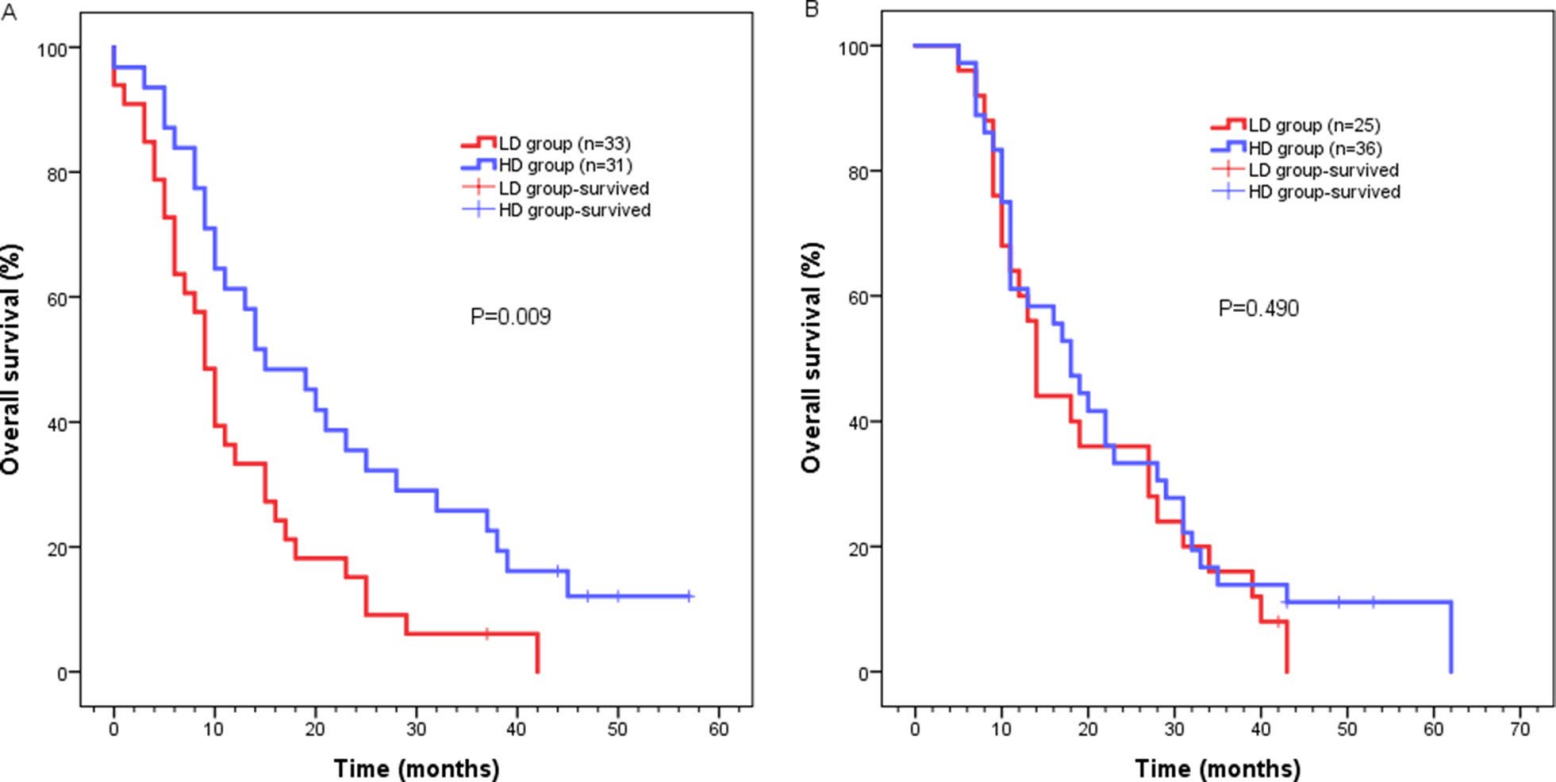
Kaplan–Meier survival analysis for LD group and HD group according to **a** re-irradiation without chemotherapy; **b** re-irradiation with chemotherapy

**Fig. 4:**
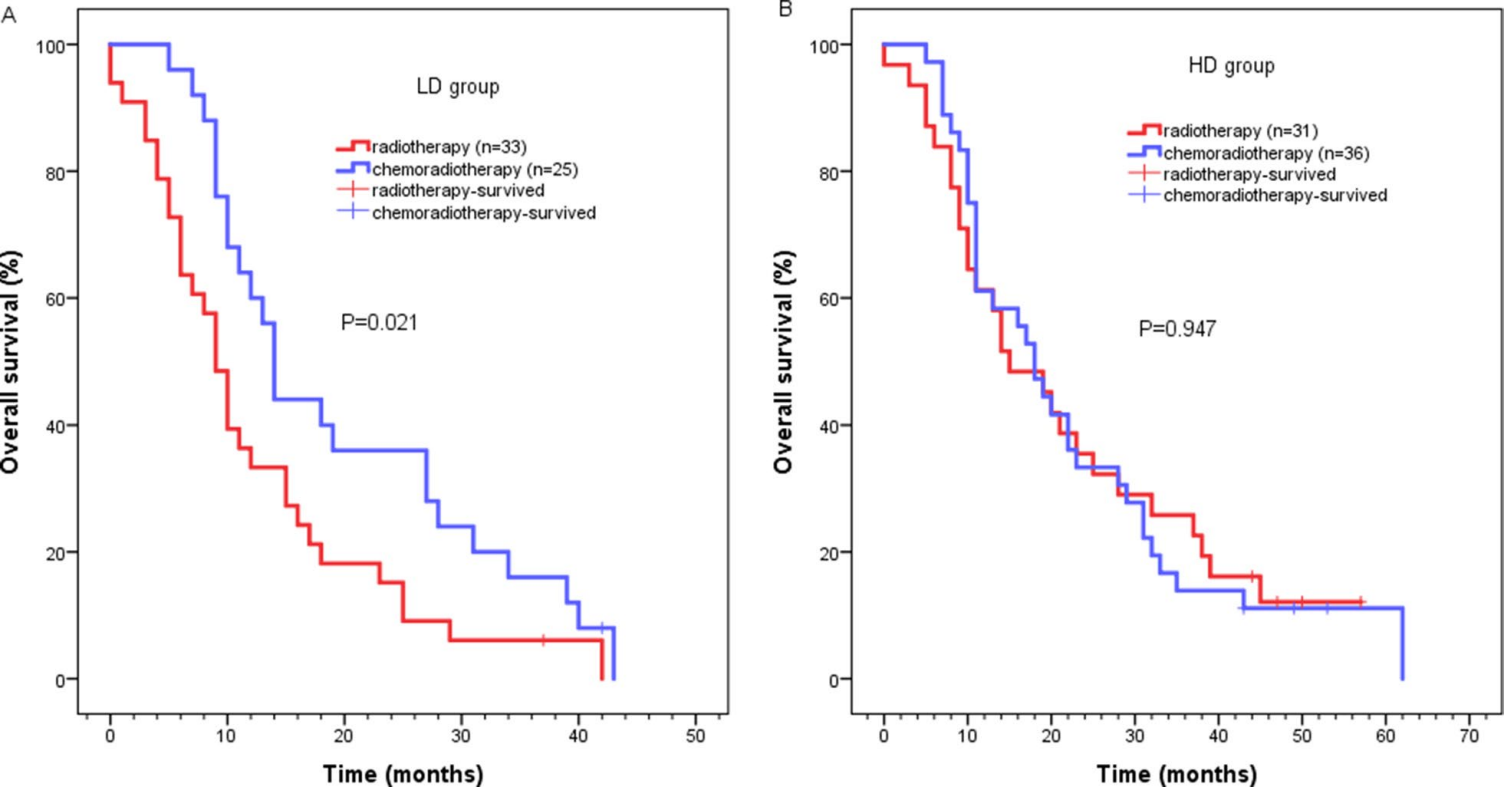
Kaplan–Meier survival analysis after re-irradiation **a** with or without chemotherapy in LD group; **b** with or without chemotherapy in HD group

The two groups of patients were then stratified based on their acceptance of chemotherapy. Patients who received radiotherapy alone in the HD group had a significantly better survival outcome compared to those who received radiotherapy alone in the LD group (median survival time: 15 months, 95% CI 7.365–22.635 vs 9 months, 95% CI 7.124–10.876, *P* = 0.009) (Fig. 3a). Median survival time of patients who received chemoradiotherapy was 18 months (95% CI 13.597–22.403) in the HD group, and 14 months (95% CI 12.378–15.622) in the LD group (*P* = 0.490). (Fig. 3b). In addition, patients in the LD group who received chemoradiotherapy had a higher median survival rate than patients who received radiotherapy alone (14 months, 95% CI 12.378–15.622 vs 9 months, 95% CI 7.124–10.876, *P* = 0.021) (Fig. 4a). Notably, the median survival time of patients with or without chemotherapy was 18 months (95% CI 13.597–22.403) and 15 months (95% CI 7.365–22.635) in the HD group, respectively (*P* = 0.947) (Fig. 4b).

### Cox regression analysis for overall sample

Univariate and multivariate analyses were conducted to determine whether the dose of re-irradiotherapy affected the patient survival (Table 2). In univariate analysis, ECOG, age, TR and re-irradiation dose were significantly associated with overall survival (OS) (*P* < 0.05 for each comparison). Similarly, multivariate analysis showed that ECOG of 0–1 (*P* = 0.048), age < 70 years (*P* = 0.028), TR > 24 months (*P* = 0.001) and re-irradiation dose of 55–60 Gy (*P* = 0.013) were independent favorable predictors of OS.

**Table 2:** Cox model analysis for 125 patients local recurrent esophageal squamous cell carcinoma following radical (chemo) radiotherapy

Variables	Number	Univariate			Multivariate		
		HR	95%CI	*P*	HR	95%CI	*P*
*Gender*		0.758	0.505–1.138	0.181			
Male	84						
Female	41						
*Age (years)*		1.519	1.046–2.208	0.028	1.472	1.010–2.144	0.044
< 70	71						
≥ 70	54						
*ECOG*		1.593	1.004–2.527	0.048	1.641	1.027–2.621	0.038
0–1	102						
2	23						
*Recurrence pattern*		1.149	0.915–1.444	0.232			
PR	67						
LNR	25						
PR + LNR	33						
*Time to recurrence (months)*		0.507	0.346–0.745	0.001	0.484	0.328–0.713	< 0.001
≤ 24	68						
> 24	57						
*Chemotherapy*		0.758	0.526–1.094	0.139			
Yes	61						
No	64						
*Re-irradiation dose*		0.626	0.432–0.908	0.013	0.638	0.439–0.927	0.018
50–54 Gy	58						
55–60 Gy	67						

*HR* Hazard ratio, *95%CI* 95% confidence interval, *ECOG* Eastern Cooperative Oncology Group Performance Status, *PR* primary recurrence, *LNR* local lymph node recurrence

### Toxicity

We observed no grade v acute or late toxicity. Radiation esophagitis was the most common acute toxicity in both groups. The incidence of radiation esophagitis was 58.6% in the LD group and 68.7% in the HD group (*P* = 0.868). Other common acute toxicities included hematological toxicity (*P* = 0.004) and gastrointestinal reaction (nausea, vomiting, loss of appetite and constipation) (*P* = 0.732). The number of patients with combined chemotherapy or radiotherapy alone who developed grade 1 radiation esophagitis, hematological toxicity, and gastrointestinal reactions were 11 vs. 8, 14 vs. 9, 5 vs. 1 in the LD group and 15 vs. 14, 8 vs. 4, 9 vs. 3 in the HD group, respectively. In LD group, there were 9 cases, 14 cases and 7 cases of grade ≥ 2 radiation esophagitis, hematological toxicity and gastrointestinal reaction in patients with chemotherapy, and 6 cases, 3 cases and 1 case of grade ≥ 2 radiation esophagitis, hematological toxicity and gastrointestinal reaction in patients with radiotherapy alone. In HD group, there were 9 cases, 20 cases and 8 cases of grade ≥ 2 radiation esophagitis, hematological toxicity and gastrointestinal reaction in patients with chemotherapy, and 8 cases, 7 cases and 2 cases of grade ≥ 2 radiation esophagitis, hematological toxicity and gastrointestinal reaction in patients with radiotherapy alone. Rare acute toxicity included radiation-induced pneumonitis, radiation tracheitis and skin reaction. Grade ≥ 2 radiation-induced pneumonitis was noted in 1 patient without chemotherapy in the HD group. A total of 11 patients, 7 patients with chemotherapy and 4 patients without chemotherapy, developed severe treatment-related toxicity, such as esophageal perforation/fistula and bleeding. The TR in 9 of the 11 patients was ≤ 24 months. Within 3 months following re-irradiation, 2 cases of bleeding and 3 cases of esophageal perforation/fistula were reported in the LD group. About 3 of the 5 cases received chemotherapy, while 1 case of bleeding and 2 cases of esophageal perforation/fistula occurred in the HD group, all these 3 cases received chemotherapy. Three months after re-irradiation, 1 case of bleeding and 2 cases of esophageal perforation/fistula occurred in the LD group, 2 of the 3 cases received chemotherapy. Severe late complications occurred 6 months after re-irradiation; 2 patients in each group had severe esophageal stenosis and underwent esophageal dilatation (Table 3).

**Table 3:** Number of patients with toxic effects in the two groups (n)

Toxicity	LD group				HD group			
	Grade 1	Grade 2	Grade 3	Grade 4	Grade 1	Grade 2	Grade 3	Grade 4
Radiation-induced esophagitis	19	13	2	0	29	14	3	0
Hematologic toxicity	23	11	6	0	12	19	8	0
Gastrointestinal reactions	6	5	3	0	12	7	3	0
Radiation-induced pneumonitis	6	0	0	0	7	1	0	0
Tracheitis	2	0	0	0	1	1	0	0
Skin reaction	0	3	0	0	0	4	0	0
Bleeding	0	0	2	1	0	0	0	1
Esophageal perforation/fistula	0	0	3	2	0	0	1	1
Esophageal stenosis	0	0	2	0	0	0	2	0

## Discussion

This study evaluated the survival outcome and toxicity of different re-irradiation doses combined with or without chemotherapy in the treatment of LRESCC. A recent study showed that, the radiotherapy dose of > 59.4 Gy after standard concurrent chemoradiotherapy (50.4 Gy), achieved complete response in patients with ESCC. This dose provided effective local control, and improved the 5-year recurrence-free survival and OS rates [15]. Moreover, there are studies indicating that a re-irradiation dose of > 50 Gy can significantly increase the survival rate of LRESCC [2, 13, 14]., Kobayashi et al*.* has demonstrated that the use of 60 Gy can be an appropriate salvage dose for LRESCC after surgery [16]. Besides, another study pointed out that patients with LRESCC with a radiation history should be given higher doses of radiotherapy [17]. These studies suggest that increasing the dose of radiotherapy for recurrent esophageal cancer may prolong the overall survival of patients.

Herein, we compared the effects of 50 Gy and 60 Gy re-irradiation doses on the survival of patients with LRESCC who had undergone radiation therapy previously. The obtained results showed 54.4% (68/125) of the patients experienced recurrence within 2 years following initial (chemo) radiotherapy, which was consistent with previous reports [2, 13, 18, 19]. It was also reported by Xu et al*.* that the 2-year survival rate and median survival time of LRESCC patients receiving ≥ 50 Gy re-radiotherapy was 37.5% and 18 months, respectively [14]. Whereas this outcome was better than our entire cohort, it was similar to the survival rates of patients in the HD group. In our study, the 2-year survival rate and median survival time for the whole cohort was 29.6% and 14 months, respectively. The 1-, 2-, or 3-year survival rate and median survival time were 61.2%, 34.3% or 19.4% and 18 months in the HD group, and 48.3%, 24.1% or 10.3% and 11 months in the LD group, respectively. Multivariate analyses clearly revealed that the dose of radiotherapy was an important factor which affects the survival rate of the patients. Whereas the median survival time of patients receiving radiation alone in the HD group was 15 months, and that of patients in the LD group was 9 months. Hence our results showed better outcome compared with previous studies [2, 13, 17]. As a result, the study clearly revealed that higher radiation dose might be more beneficial for tumor control. We, therefore, recommend higher doses of re-radiation for patients with recurrent ESCC after radiotherapy.

Effective local tumor control will improve the survival rate of patients [20]. To date, few studies have reported the rate of local control following re-irradiation therapy in patients with LRESCC. Although previous studies have reported that higher radiation doses can improve the local control rate of esophageal cancer and survival rate [21, 22]. In the present study, the 3-year locoregional control rate in the LD group and HD group was 3.4% and 14.9%, respectively. The lymphatic drainage was not included in the treatment strategy hence the rates were low.

Studies have demonstrated positive effect of chemotherapy in the initial treatment of ESCC [23]. However, data on the role of chemotherapy and re-irradiation therapy of ESCC is scant however only few studies have shown the role of chemotherapy and re-radiotherapy in recurrent ESCC. Chen et al*.* reported that the 1-, 2- or 3-year survival rate of 36 LRESCC patients receiving re-irradiation with concurrent chemotherapy (paclitaxel + cisplatin) was 51.7%, 21.4% or 12.2%, respectively [2]. While Katano et al*.* reported that the median survival time for 6 patients who underwent concurrent chemotherapy (nedaplatin and tegafur) with re-radiotherapy was 13.6 months [10]. In a stratified analysis, we found that chemotherapy coupled with radiotherapy increased the survival rate. In LD group, the median survival time following chemoradiotherapy was 14 months, which was consistent with results from a previous study [10]. However, in the HD group, chemoradiotherapy did not improve the survival rate when compared to radiotherapy alone. Our results suggest that chemotherapy combined with re-radiotherapy can increase the survival rate of LRESCC patients, when patients are exposed to lower radiation doses.

Comparison of our experience with available data is limited. We provide toxicity as much as possible for reference. In the present study, acute radiation esophagitis was the most common toxicity in the whole cohort. The incidence of esophagitis in the HD group was significantly higher than in the LD group (68.7% vs 58.6%, *P* = 0.868). Most importantly either the LD group or the HD group, chemotherapy may increase the incidence of radiation esophagitis. Previous studies have shown that the incidence of severe acute radiation esophagitis in patients receiving thoracic radiotherapy was 15–25% [24]. The incidence of grade ≥ 3 acute esophagitis in both groups was low, hematological toxicity and gastrointestinal reactions were also common. More than two-thirds of patients who developed hematological toxicity or gastrointestinal reactions has received chemotherapy in LD group or HD groups. The chemotherapy increased the risk of hematological toxicity and gastrointestinal reactions, especially grade ≥ 2. Moreover, the incidence of radiation pneumonitis was lower in the present study compared to previous studies [25]. Grade 1–2 radiation pneumonitis occurred in six (10.3%) and eight (11.9%) patients in the LD and HD groups, respectively, with controllable symptoms. We also noticed severe complications within 3 months following re-irradiation. A total of 11 patients had severe treatment-related toxicity, such as bleeding, esophageal perforation, and esophageal fistula, eight of which were in the LD group. This may be related to the TR of ≤ 24 months in most patients in the LD group (36/58, 62.1%). Severe late complications, such as esophageal stenosis and dysphagia were effectively resolved by esophageal dilation. These results revealed that higher re-irradiation dose may be a safe treatment option. Given that low-dose radiotherapy combined with chemotherapy has similar survival results with higher-dose radiotherapy alone (median survival time: 14 months vs 15 months). It is noted that chemotherapy can results in the increased risk of hematological toxicity and gastrointestinal reactions, higher-dose radiotherapy alone should be prior to low-dose radiotherapy combined with chemotherapy. If higher dose re-radiation cannot be executed, we recommend low-dose re-radiation combined with chemotherapy which will be more conducive to improving long-term survival. The LD alone may be suitable for patients with palliative care. For patients with TR ≤ 24 months, the possibility of severe treatment-related toxicity needs to be carefully monitored.

## Conclusion

Taken together, a higher re-irradiation dose (55–60 Gy) for LRESCC patients who had received radiation therapy may yield better long-term survival time, and should be the first choice. More data are needed to verify whether higher re-irradiation dose combined with chemotherapy can further improve the survival benefit. Re-irradiation with lower doses (50–54 Gy), combined with chemotherapy can also increase the survival time of LRESCC patients, if a higher re-irradiation dose (55–60 Gy) cannot be implemented. For patients with a short recurrence time (TR ≤ 24 months), it is important to monitor the risk of severe toxicity.

## Data Availability

All data generated or analyzed during this study are included in this article.

## Abbreviations

LRESCC: Locally recurrent esophageal squamous cell carcinoma
LD group: Low-dose group
HD group: High-dose group
ESCC: Esophageal squamous cell carcinoma
ECOG: Eastern Cooperative Oncology Group Performance Status
3D-CRT: Three-dimensional conformal radiotherapy
IMRT: Intensity modulated radiotherapy
CT: Computed tomography
BED: Biologically effective dose
RR: Response rate
GTV: Gross tumor volume
GTVnd: Gross tumor volume of the metastatic lymph nodes
PTV: Planning target volume
OS: Overall survival
RECIST 1.1: Response Evaluation Criteria in Solid Tumors Version 1.1
CTCAE v4.0: Common Terminology Criteria for Adverse Events version 4.0
TR: Time to recurrence
LNR: Local lymph node recurrence
PR: Primary recurrence
CI: Confidence interval
